# Thrombocytopaenia in pregnant women with malaria on the Thai-Burmese border

**DOI:** 10.1186/1475-2875-7-209

**Published:** 2008-10-15

**Authors:** Saw Oo Tan, Rose McGready, Julien Zwang, Mupawjay Pimanpanarak, Kanlaya Sriprawat, Kyaw Lai Thwai, Yoe Moo, Elizabeth A Ashley, Bridget Edwards, Pratap Singhasivanon, Nicholas J White, François Nosten

**Affiliations:** 1 Shoklo Malaria Research Unit (SMRU), PO Box 46 Mae Sot, Tak, 63110, Thailand; 2 Mahidol-Oxford Tropical Medicine Research Unit (MORU), Mahidol University, Bangkok, 10400, Thailand; 3 Centre for Clinical Vaccinology and Tropical Medicine, Churchill Hospital, Oxford, OX3 7LJ, UK

## Abstract

**Background:**

Haematological changes associated with malaria in pregnancy are not well documented, and have focused predominantly on anaemia. Examined here is thrombocytopaenia in pregnant women infected with *Plasmodium falciparum *or *Plasmodium vivax *in a low transmission area on the north-western border of Thailand.

**Methods:**

In this observational study we reviewed the platelet counts from routine complete blood count (CBC) in a cohort of healthy and malaria infected Karen pregnant women attending weekly antenatal clinics. A platelet count of 75,000/μL was the threshold at 2 standard deviations below the mean for healthy pregnant women used to indicate thrombocytopenia. Differences in platelet counts in non-pregnant and pregnant women were compared after matching for age, symptoms, malaria species and parasitaemia.

**Results:**

In total 974 pregnant women had 1,558 CBC measurements between February 2004 and September 2006. The median platelet counts (/μL) were significantly lower in patients with an episode of falciparum 134,000 [11,000–690,000] (N = 694) or vivax malaria 184,000 [23,000–891,000] (N = 523) compared to healthy pregnant women 256,000 [64,000–781,000] (N = 255), P < 0.05 for both comparisons. *Plasmodium falciparum *and *P. vivax *caused a 34% (95% CI 24–47) and 22% (95% CI 8–36) reduction in platelet count, respectively. Pregnant compared to non pregnant women were at higher risk OR = 2.27 (95%CI 1.16–4.4) P = 0.017, for thrombocytopaenia. Platelets counts were higher in first compared with subsequent malaria infections within the same pregnancy. Malaria associated thrombocytopaenia had a median [range] time for recovery of 7 [2-14] days which did not differ by antimalarial treatment (P = 0.86), or species (P = 0.63) and was not associated with active bleeding.

**Conclusion:**

Pregnant women become more thrombocytopenic than non-pregnant women with acute uncomplicated malaria. Uncomplicated malaria associated thrombocytopaenia is seldom severe. Prompt antimalarial treatment resulted in normalization of platelet counts within a week.

## Background

Pregnancy increases susceptibility to malaria[1] and is associated with profound alterations in the fibrinolytic and coagulation systems [2]. While normal pregnancy produces physiological changes resulting in a procoagulant effect (to minimize intrapartum blood loss) [2], malaria [3], in particular severe malaria caused by *Plasmodium falciparum*, can cause profound anaemia, thrombocytopaenia, activation of the coagulation cascade and rarely disseminated intravascular coagulopathy [4]. The extent of these changes in cases of uncomplicated malaria in pregnancy has not been previously described.

## Methods

The Shoklo Malaria Research Unit (SMRU) has been conducting antenatal clinics (ANCs) in refugee camps on the Thai-Burmese border since 1986 and in clinics for migrant women since 1998. ANCs provide pregnant women with early malaria detection, by weekly blood smear, and prompt treatment of malaria to prevent maternal death. Malaria transmission is low and seasonal in the area and there is a high prevalence of multi-drug resistant strains of *Plasmodium falciparum *[5] and chloroquine sensitive *P. vivax*. There are no effective drugs for prophylaxis in pregnancy and randomized controlled trials with bed nets and skin repellents given specifically for pregnant women, failed to show a significant preventive effect [6,7]. Attendance at ANC is voluntary and all women are encouraged to attend weekly. Since the inception of this programme there have been no maternal deaths from malaria in women who attend weekly, where previously malaria related maternal mortality was of the order of 1,000/100,000 live births [8]. Anaemia is monitored every two weeks and prophylactic and treatment doses of ferrous sulphate and folic acid are provided until delivery. Women are encouraged to come and deliver under supervision in the SMRU facilities. Complicated deliveries requiring Caesarean section are referred to Mae Sot hospital (one hour drive).

Women with malaria are routinely asked about symptoms and assessed for spleen and liver size. All cases with positive malaria smears are treated: falciparum or mixed infections receive quinine sulphate 10 mg/kg three times a day for seven days or artesunate 2 mg/kg once per day for seven days, where possible, in combination with clindamycin 300 mg three times daily for seven days. *Plasmodium vivax *infections are treated with 25 mg base/kg of chloroquine given over three days (10, 10, 5 mg base/kg/day).

Since 2004, it has been possible to offer pregnant women a complete blood count (CBC) as part of routine care during a malaria attack and (in some sites) at booking consultation. CBC is repeated within the same patient if clinically indicated e.g. to monitor thrombocytopaenia [9]. Women with reduced platelet counts have them rechecked at 48 hours and the CBC is only repeated again (at day 7 and 14) if they have not normalized. As part of routine antenatal care, blood pressure is checked on admission, at any episode of malaria or other illness, and at 28, 32, 34 and 36 weeks and then weekly until delivery.

In order to compare CBC data in pregnant and non-pregnant women, using the same automated haematology analyzer as used for pregnant women, data from 71 non-pregnant women of child bearing age, median [range] 25 years [15–45] years) with symptomatic uncomplicated falciparum malaria from the same population were analysed [10]. These were compared to a selected group of 108 pregnant women febrile with acute uncomplicated falciparum malaria, and the first infection for the pregnancy of any species of malaria Pregnant women with asymptomatic infection detected by the active weekly screening were excluded as they were not thought to be comparable.

### Laboratory samples and processing

Blood smears (thin and thick films) were prepared using Giemsa staining and were read for 200 fields before being declared negative. Parasite counts were reported per 500 white blood cells (WBC) and for counts above 1,000 parasites per 500 WBC by the percentage of infected red cells (RBC). All stages of the parasites were recorded (asexual and gametocytes).

A 2 ml sample of venous blood was placed in an EDTA tube, refrigerated and transported on ice to SMRU Mae Sot laboratory, where the complete blood count was determined using a Sysmex pocH-100i automated haematology analyzer. Quality control of the Sysmex pocH-100i was determined on a daily basis by analysis of three different manufacturer-provided samples with known cell counts.

### Definitions

Thrombocytopaenia in pregnancy was defined by a platelet count lower than 75,000/μL. This is the value (rounded up to the nearest 5,000) that falls two standard deviations below the mean platelet count in healthy Karen pregnant women (mean 198,000/μL, SD: 62,500, min-max [22,000–540,000], n = 723) from a previously published cohort study from the same area [11]. This value was used for three reasons: 1) the definition used for thrombocytopaenia in pregnant women varies in the literature e.g. 115,000/μL [12-14], 150,000/μL [15] and 75,000/μL [9], 2) pregnancy itself causes platelet count to decrease[9] and 3) the original cohort included healthy pregnant women of all gestations[11] and there was no significant difference with platelet count by trimester.

Healthy women were defined as pregnant women without malaria or other febrile illness at booking consultation. Asymptomatic malaria was defined by the presence of asexual forms of *P. falciparum *or *P. vivax *on the microscopic examination of the peripheral blood in a pregnant women with no elevation of temperature (aural < 37.5°C), no history of fever or any of the following symptoms: headache, dizziness, joint pain, anorexia, nausea, spontaneous bleeding. Symptomatic malaria was defined as the presence of an elevated temperature (aural ≥ 37.5°C), or a history of fever in the previous two days, with one or more of the above symptoms in a pregnant women together with asexual forms of *Plasmodium *on the microscopic examination of the peripheral blood. Uncomplicated malaria was defined by the absence of signs of severity (WHO criteria[16]) and uncomplicated hyperparasitaemia by 4% parasitized red blood cells or more in a patient with no sign of severity. Hypertension was defined as a blood pressure of 140/90 mmHg or more with or without proteinuria. Eclampsia was defined as hypertension and proteinuria detected for the 1^st ^time after 20 weeks gestation.

Estimated gestational age (EGA) at the time of CBC was determined from ultrasound (US) dating of the pregnancy. As part of routine ANC pregnant women had a US scan at booking and at 18 weeks. Splenomegaly was recorded as present when the spleen was palpable below the costal margin and was measured in cm. Hepatomegaly was recorded as present when the liver was palpable below the costal margin in the mid-clavicular line.

### Statistical analysis

Continuous normally distributed data were described by the mean (standard deviation) and non-normally distributed data by the median [range]. Percentages were given for categorical data. Categorical data were compared using the Chi-square test or by Fisher's exact test, as appropriate. Student's t-test was used to analyze means, the Mann-Whitney test was used to analyze medians. The Wilcoxon rank sum test was performed on continuous data with a skewed distribution for paired analysis to determine whether there were significant differences of parasitaemia and haematological counts after or between episodes of malaria. Forward and backward stepwise logistic regression was used to assess the relationship with thrombocytopaenia, while controlling for demographic characteristics (age), and potential confounding factors significant on univariate analysis (parasitaemia, gestational age at time of CBC, symptoms, fever and spleen size as appropriate). Age, and log transformed parasitaemia were treated as a continuous variables and the others (fever: temperature ≥ 37.5°C, pregnancy status) as dichotomous variables. Data were analyzed using EpiInfo version 6, and SPSS version 14 for Windows (SPSS Inc).

## Results

**Figure 1:**
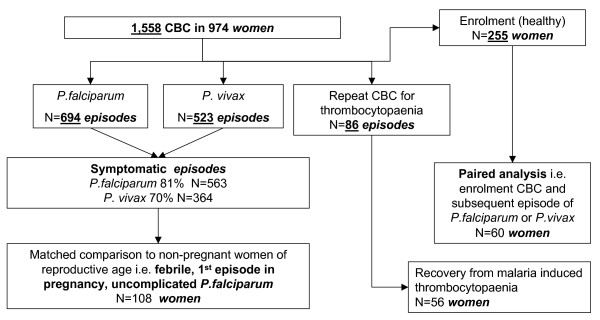
Flow diagram of the various stages of analysis showing the number of CBCs available in terms of malaria episodes or in particular women.

**Figure 2:**
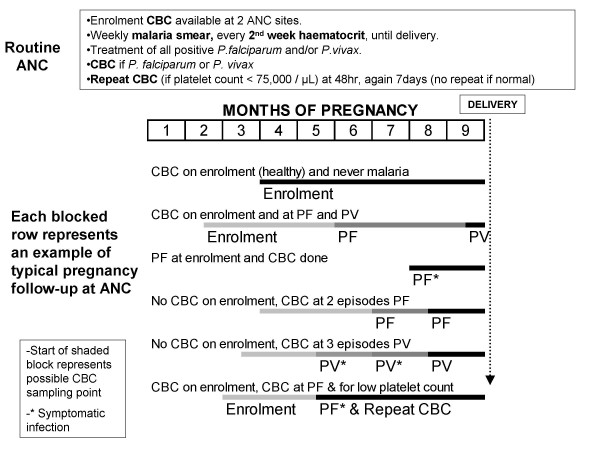
Schematic diagram of typical examples of pregnancy follow-up in individual patients and when CBC samples could have been collected.

Between February 2004 and September 2006, 974 pregnant women had 1,558 CBC measurements. CBC results were available for 255 healthy women from their antenatal booking consultation (all these women had a negative blood smear), 694 acute *P. falciparum *episodes, 523 *P. vivax *episodes, and 86 were repeat measurements following a report of thrombocytopaenia (Figure 1). There were more episodes than there were women in the cohort. Figure 2 presents possible scenarios of CBC measurements within a single pregnancy and highlights the considerable variation in the possible sequence and gestation of malaria episodes (Figure 2).

Co-infections of falciparum with vivax malaria (n = 49) showed similar haematological changes to falciparum monoinfections but were excluded from analysis in order to focus on the effects of *P. falciparum *and *P. vivax *separately.

**Figure 3:**
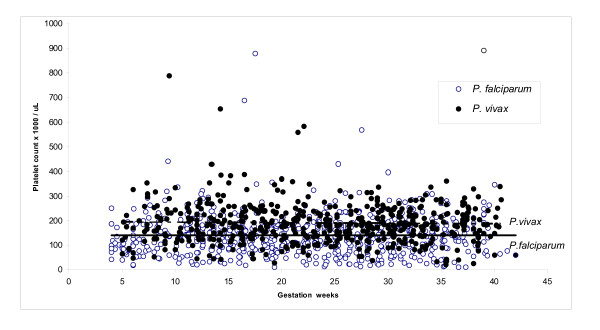
The relationship between gestational age and platelet count for *Plasmodium falciparum *and *P. vivax *infection.

The proportion of uncomplicated malaria episodes was 97.9% (1,191/1,217) and 2.1% (26/1,217) were hyperparasitaemic episodes (≥ 4% RBC parasitized). The three groups (Healthy, first episode *P. falciparum *and first episode *P. vivax*) were comparable for age, gravidity and parity at the time of the first CBC (Table 1). Estimated gestational age at the time of the CBC was significantly lower (P = 0.001) in the healthy group as this was done at the booking consultation and malaria is more likely to occur in the 2nd trimester of pregnancy. The gestation at each episode of malaria, *P. falciparum *or *P. vivax*, was compared with the platelet count and no significant association was evident (Spearman Rho 0.012 and 0.001; P = 0.0756 and P = 0.980, respectively) (Figure 3). Overall 2.7% (26/974) of women required blood transfusion during pregnancy or immediately post-partum of whom 85% (22/26) were transfused for malaria related symptomatic anaemia.

**Table 1:** Characteristics of Karen and Burmese women at the time of their first CBC in pregnancy

	Healthy	*Plasmodium falciparum*	*Plasmodium vivax*
	N = 255	N = 425	N = 294
Maternal Age, yrs	25	25	25
	[15–46]	[14–43]	[15–42]
EGA (weeks)	14.0	19.6	22.1
	[4.3–39.4]	[4–41.2]	[4–40]
Gravidity	3 [1–14]	3 [1–13]	3 [1–11]
Parity	1 [0–10]	2 [0–11]	1 [0–9]
Proportion primigravidas % (n)	26 (65/255)	24 (103/425)	28 (81/294)

Data are median and [range] unless otherwise indicated.

No difference in primips proportions (P = 0.605).

As eclampsia is typically associated with thrombocytopaenia we reviewed the 1.3% (13/964) of women with hypertension in pregnancy individually. As the CBC for malaria was measured earlier in pregnancy than the hypertension developed, particularly for women with a diagnosis of eclampsia (n = 3), and these women were not thrombocytopenic their CBC results were not excluded from the malaria analysis.

### Malaria species, symptoms, hepatosplenomegaly and parasitaemia

On admission, falciparum cases had significantly higher parasitaemia, days of fever, proportion febrile or symptomatic, and higher rates of enlarged spleen and liver, compared to vivax cases (Table 2). The proportion of episodes of malaria with any symptoms, history of fever in the last two days or fever on admission was 76.2% (927/1,217) and significantly higher for *P. falciparum *(81%, 563/694) than for *P. vivax *(70%, 364/523) episodes, P = 0.001. (Table 2). More women with splenomegaly had thrombocytopaenia than those without a palpable spleen: 25.3% (19/75) *vs *11.2% (124/1,106), P = 0.001. Splenomegaly was associated with a 2.7 (95%CI: 1.5–4.7) fold risk of thrombocytopaenia. The number of women who had spontaneous bleeding at the time of the malaria episode such as nose bleeds or gum bleeding was very small, and not significantly associated with thrombocytopaenia (OR = 1.95, 95%CI 0.65–5.82, P = 0.226). There was no difference in the proportion of reported antepartum haemorrhage (APH) in women who had any episode of thrombocytopaenia, 23% (5/22), compared to those who had none, 18% (114/622, P = 0.60). The geometric mean parasitaemia was significantly lower in afebrile compared to febrile patients (485/μL and 9,957/μL, respectively, P = 0.001), and asymptomatic episodes compared to symptomatic episodes (218/μL and 1,385/μL, respectively, P = 0.001). Geometric mean parasitaemia of *P. falciparum *was higher than that of *P. vivax*: 2,176/μL and, 273/μL respectively, P = 0.001.

**Table 2:** Malaria episode details and symptoms in Karen and Burmese pregnant women according to species

	*Plasmodium falciparum*	*Plasmodium vivax*
Geometric mean [range] parasitaemia/μL	2,288 [6–569,634]	273 [1–82,934]
Median episode number	1 [1–9]	1 [1–10]
Median EGA, weeks	22.4 [4–42]	24.3 [5–40.6]
Proportion with splenomegaly	10.5 (66/628)	2.0 (8/399)
Median [range] spleen size, cm	3 [1–13]	3 [1–4]
Proportion with hepatomegaly	15.7 (99/630)	5.8 (23/399)
Median [range] liver, cm	3 [1–9]	2 [1–5]
Proportion of asymptomatic episodes	18.9 (131/694)	30.4 (159/523)
Proportion febrile (admission)	27.6 (180/652)	10.8 (52/482)
Proportion women with a history of fever	58.6 (407/694)	28.5 (149/523)
Median days of fever	2 [1–30]	2 [1–9]
Proportion headache	58.0 (393/678)	45.7 (233/510)
Proportion dizziness	34.9 (237/679)	34.9 (178/510)
Proportion joint pain	38.1 (259/679)	34.7 (177/510)
Proportion anorexia	35.8 (243/678)	23.7 (121/510)
Proportion nausea	26.7 (181/679)	22.5 (115/510)

Please note that headache, dizziness, joint pain, anorexia and nausea symptoms were not recorded for 16 and 13 episodes of falciparum and vivax, respectively.

### Effect of malaria on platelet count

Platelet counts (median, [range]/μL) in episodes of malaria infection, *P. falciparum *134,000 [11,000–690,000] and *P. vivax *184,000 [23,000–891,000]/μL, were significantly lower compared to healthy controls, 256,000 [64,000–781,000]/μL, P < 0.05 for both comparisons. The platelet counts in falciparum cases were significantly lower than in vivax cases, P < 0.05. The proportion of women with thrombocytopaenia, were healthy 0.8% (2/255), falciparum 17.7% (123/694) and vivax 5.0% (26/523), P < 0.05 for both comparisons.

**Figure 4:**
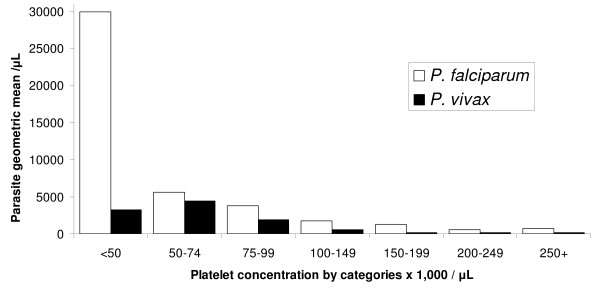
The relationship between parasitaemia and platelet count for *Plasmodium falciparum *and *P. vivax *infection in pregnancy.

There were no cases of severe (< 10,000/μL) thrombocytopaenia (lowest platelet count = 11,000/μL in the falciparum group). In the remaining cases, there were 51.7% (78/151) counts from 10,000 to < 50,000/μL and 48.3% (73/151) count from 50,000 to < 75,000/μL. There was no association between species of infection and having a count of 10,000 to < 50,000/μL or 50,000 to < 75,000/μL (P = 0.866, data not shown). A parallel trend was observed between parasitaemia and thrombocytopaenia for falciparum and vivax cases (Figure 4). The proportion of asymptomatic malaria episodes with thrombocytopaenia was 4.0% (6/151). These cases were all *P. falciparum *infections.

### Quantification of impact of malaria infection on platelet count in individual patients

There were 60 women who had their first CBC at enrolment (healthy) and then a subsequent CBC for a malaria episode (*P. falciparum *= 24 or *P. vivax *= 36). Platelet counts all decreased with malaria, the impact of *P. falciparum *being significantly greater than that of *P. vivax *(Table 3). The median thrombocytopenic effect of malaria species was estimated from baseline (healthy) counts as a reduction of 34%, (95% CI: -24 to -47) for falciparum cases and a reduction of 22% (95% CI: -16 to -29) for vivax cases, respectively (Table 3).

**Table 3:** Paired analysis of haematological parameters: booking consultation (healthy) and at the first episode of malaria

**Species (N)**		**Parameter**	**Median**	**min**	**max**	P*
*P. falciparum *(N = 24)	Platelet	Baseline (healthy)/μL	256,000	115,000	419,000	< 0.001
		Reduction from baseline/μL	-86,000	-305,000	-5,000	
		% difference (95%CI)	-34 (-24 to -47)%			
	Haemoglobin	Baseline (healthy) g/dL	11.2	9.9	14.2	0.005
		Reduction from baseline g/dL	-0.3	-3.3	1.9	
		% difference (95%CI)	-3 (-2 to -4)%			
	WBC	Baseline (healthy)/μL	9,900	4,500	13,900	0.070
		Reduction from baseline/μL	-1,250	-6,400	3,100	
		% difference (95%CI)	-13 (-9 to -18)%			
*P. vivax *(N = 36)	Platelet	Baseline (healthy)/μL	242,000	101,000	438,000	< 0.001
		Reduction from baseline/μL	-52.5	-376	129	
		% difference (95%CI)	-22 (-16 to -29)%			
	Haemoglobin	Baseline (healthy) g/dL	11.4	8.9	12.9	0.031
		Reduction from baseline g/dL	-0.6	-2.4	1.2	
		% difference (95%CI)	-5 (-4 to -7)%			
	WBC	Baseline (healthy)/μL	8,900	1,300	24,00	< 0.001
		Reduction from baseline/μL	-750	-16,600	11,800	
		% difference (95%CI)	-8 (-6 to -12)%			

*P paired analysis of reduction from baseline

### Effect of repeated episodes of malaria in pregnancy on thrombocytopaenia

The proportion of women who had multiple episodes of malaria was 49% (381/781). A second episode occurred in 27% (214/781), a third in 11% (85/781), and 4 or more episodes in 10% (82/781). In falciparum malaria cases, the proportion of women with thrombocytopaenia was higher in the first episode 22%, (80/356), compared to later episodes of *P. falciparum *13% (43/338), OR = 2.0 (95%CI 1.3–3.0), P = 0.001, and this was also observed for vivax malaria: 11% (30/279) *versus *3% (6/238), respectively, OR = 2.9 (95%CI 1.2–7.4), P = 0.018.

### Recovery from thrombocytopaenia following treatment for malaria in pregnancy

**Figure 5:**
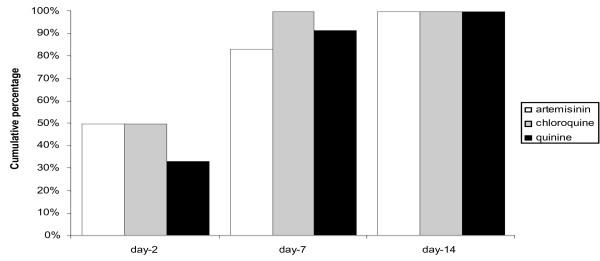
**Cumulative percent of thrombocytopaenia recovery by day platelet count was measured and drug treatment. **□artemisinin, ▩ chloroquine (for *P. vivax *only), ■quinine.

Data on women who had malaria and a repeat CBC due to thrombocytopaenia were limited to 56 women. The median [range] time for recovery from thrombocytopaenia was 7 [2-14] days (N = 56) and this did not differ between species (P = 0.632). The median time for recovery from thrombocytopaenia did not vary with the drug (quinine, artesunate, chloroquine) used for treatment (P = 0.858) (Figure 5).

### Thrombocytopaenia in pregnant and non-pregnant reproductive age women

CBC data from 71 non-pregnant women of child bearing age, median [range] 25 years [15–45] years) with uncomplicated falciparum malaria from the same population [10] were compared to 108 pregnant women whose malaria episode was febrile, uncomplicated falciparum, and the first of any species of infection for the pregnancy. A significantly greater proportion of pregnant women were thrombocytopenic 47% (51/108) than non-pregnant women 26% (18/70, P = 0.004). There was no difference in the proportion of women with splenomegaly between pregnant 7.4% (8/108), and non-pregnant women, 12.7% (9/71); P = 0.24. Pregnant women had significantly higher geometric mean parasitaemia than non-pregnant women: 13,810 [10–569,634] *versus *6,628 [53–176,594]/μL, P = 0.012. A multivariate analysis controlling for independent factors such as the parasitaemia found that both pregnancy (OR = 2.27, 95%CI 1.16–4.4, P = 0.017) and parasitaemia (OR = 1.64, 95%CI 1.12–2.39, P = 0.011) were significant independent risk factors for thrombocytopaenia.

### Pregnancy outcomes

Overall 76.0% (740/974) of women had documented deliveries, 5.7% (56/974) aborted and 18.3% (178/974) of women were lost to follow up before the pregnancy outcome was known mostly because they moved from the study area. Twins accounted for 0.7% (5/740) of deliveries. There were 1.5% (11/740) of women who had a caesarean section, all for obstetric indications and none as a result of thrombocytopenic haemorrhage. There were 69.5% (511/735) of singletons weighed in the first 3 days of life. The mean ± SD [range] birth weight was 2,868 ± 458 [1,200–4,200] g and mean gestational age 39.5 ± 1.6 [28.0–44.4] weeks. There was no significant difference in mean birth weight or gestational age at delivery in women with malaria who had or did not have thrombocytopaenia reported during pregnancy (data not shown). The rate of post-partum haemorrhage (PPH), 2.0% (10/503), was low. PPH in women with malaria occurring less than 5 days before delivery compared to ≥ 5 days was 2.9%% (2/69) *vs *1.2% (6/501), P = 0.25. The two women with PPH and malaria both had *P. vivax *infection and both were placenta negative having started treatment 1 day and 4 days prior to delivery.

## Discussion

The observational data presented here demonstrates, as others have for non-pregnant patients, the reduction in platelet count associated with both falciparum and vivax malaria [3,17-20]. In this cohort, approximately one in five episodes of falciparum malaria and one in 20 episodes of vivax malaria were associated with significant thrombocytopaenia. The degree of thrombocytopaenia associated with malaria in pregnancy is likely to have been underestimated. The weekly screening for malaria in the ANC[8] in this setting is a very active process of case finding and it will often detect infection before women are symptomatic. This is unlike the standard case management method described by WHO as one of the package of interventions for control of malaria in pregnancy[21] where women arrive to the clinic because they have symptoms and are treated.

Reduced platelet counts during malaria infection result from platelet activation, splenic pooling, and a decreased platelet life-span to 2–3 days (from normal 7–10 days) [4,17]. The role of immunological factors remains uncertain as the reduction in platelet count is directly proportional to disease severity, and recovers promptly with recovery from the infection[22]. Pregnancy itself can also cause thrombocytopaenia for reasons that are not fully understood [9]. The rate of platelet recovery following initiation of treatment for malaria is reported variably as between 4 to 10 days but differs with the severity of malaria and antimalarial treatment prescribed [23-25]. In pregnant women, a median time to platelet recovery of seven days was observed. Platelet recovery time was not affected by the type of antimalarial drug treatment. Thrombocytopaenia in malaria was usually asymptomatic. Very few women reported spontaneous bleeding at the time of acute infection and bleeding was not significantly associated with platelet counts. None of the malaria cases were affected by autoimmune thrombocytopaenia of pregnancy [9] as 100% of women showed recovery after treatment. The birth weight and gestation were not significantly affected by these episodes or thrombocytopaenia.

In this population of pregnant women with low premunition (similar to non-immune travelers), there was an approximate reduction in platelet count by one third in *P. falciparum *and one fifth in *P. vivax *infections. Another important finding was that thrombocytopaenia, although uncommon, was also seen in asymptomatic malaria infected women with *P. falciparum*. This has recently been reported in children in Nigeria [26].

Nearly 50% of women in this cohort had subsequent malaria infections. The risk of thrombocytopaenia was greatest in the first infection compared to later infections but one cannot determine whether this is a protective effect gained by a single infection or a by product of intensive weekly screening where parasitaemia may be detected earlier and before the woman becomes symptomatic.

Does this level of thrombocytopaenia put women with malaria at the time of delivery at risk of post-partum haemorrhage? Spontaneous bleeding is uncommon unless the platelet count falls below 10,000/μL [27] when any patient would be treated with platelet transfusion. This is in keeping with the observations reported here of no association with APH or spontaneous bleeding with thrombocytopaenia. Adequate haemostasis with spontaneous vaginal delivery and caesarean section can take place at concentrations above 50,000/μL [14]. Where facilities exist obstetricians have a tendency to transfuse platelets at concentrations below 50,000/μL when elective caesarean section is planned. In this cohort, 52% of thrombocytopaenia associated with malaria occurred with platelet counts below 50,000/μL, but none were below 10,000/μL. In resource rich settings, obstetric practice is pre-emptive and few women deliver with platelet counts known to be between 10–50,000/μL without platelet transfusion. The risk of haemorrhage associated with normal vaginal delivery in women with a platelet count in this range is unknown. So the risk of post-partum haemorrhage for the 50% of episodes of thrombocytopaenia that are below 50,000/μL cannot be answered conclusively.

Clearly treatment with antimalarials leads to platelet recovery but this takes closer to seven days than 48 hours. This implies it would probably be detrimental to try to postpone labour in women with treated uncomplicated malaria at term to allow for platelet recovery with treatment. This may well not apply to severe malaria where platelet counts are likely to be more severely affected, and there is a high risk of both fetal and maternal death[4].

The women presented here reside in an area of low and unstable malaria transmission where acute symptomatic malaria can occur in women of all gravida [8] and placental malaria at delivery is uncommon [28]. In sub-Saharan Africa, adverse affects in primigravida are pronounced and high rates of placenta malaria 26% (5–52%) are reported [29-31]. However the change in platelet counts in pregnant women with chronic infections has not been determined. An attempt was made to quantify blood loss at delivery in women with placental malaria in Tanzania but it was not possible to measure platelet counts [32]. An observational study reported a greater risk of PPH in malaria transmission areas of PNG [33]. Ideally a trial that could account for malaria infection (peripheral and placenta), platelet count and post-partum haemorrhage in a higher transmission area is needed to positively determine if the thrombocytopaenic effects of malaria are directly associated with PPH.

Falciparum and vivax malaria adversely affect platelet count in pregnancy and pregnant women are more susceptible to thrombocytopaenia than non-pregnant women. The effect of chronic malaria infection in pregnancy on thrombocytopaenia is unknown. Nevertheless treatment results in recovery from thrombocytopaenia and where efforts to prevent malaria fail, prompt detection and treatment, particularly in the last week before delivery should be provided.

## Competing interests

The authors declare that they have no competing interests.

## Authors' contributions

SOT, RM, EAA, MPJ, KS, KLT and YM participated in the clinical work and data compilation. RM, BE and FN conceived the need to review clinical records. SOT, KLT, YM and JZ performed the data management and JZ the statistical analysis. SOT, RM, JZ, EAA, BE, PS, NJW, and FN participated in drafting the manuscript. All authors read and approved the final manuscript.
